# A Novel Approach: Evaluating ChatGPT's Utility for the Management of Thyroid Nodules

**DOI:** 10.7759/cureus.47576

**Published:** 2023-10-24

**Authors:** Ekin Y Köroğlu, Sevgül Fakı, Nagihan Beştepe, Abbas A Tam, Neslihan Çuhacı Seyrek, Oya Topaloglu, Reyhan Ersoy, Bekir Cakir

**Affiliations:** 1 Endocrinology and Metabolism, Ankara City Hospital, Ankara, TUR; 2 Endocrinology and Metabolism, Ankara Yıldırım Beyazıt University School of Medicine, Ankara, TUR

**Keywords:** chatgpt, thyroid, thyroid cancer, artificial intelligence, thyroid nodule

## Abstract

Background and objective

Artificial intelligence (AI) applications such as Chat Generative Pre-Trained Transformer (ChatGPT) created by OpenAI, which represent the revolutionary aspects of today's technology, have benefitted professionals in many fields and society at large. In this study, we aimed to assess how effective is ChatGPT in helping both the patient and the physician manage thyroid nodules, a very common pathology.

Methods

Fifty-five questions frequently asked by patients were identified and asked to ChatGPT. Subsequently, three cases of thyroid nodules were progressively presented to ChatGPT. The answers to patient questions were scored for correctness and reliability by two endocrinologists. As for the cases, diagnostic and therapeutic approaches provided by ChatGPT were analyzed and scored by two endocrinologists for correctness, safety, and usability. The responses were evaluated by using 7-point Likert-type scales designed by us.

Results

The answers to patient questions were found to be mostly correct and reliable by both raters (Rater #1: 6.47 ± 0.50 and 6.27 ± 0.52; Rater #2: 6.18 ± 0.92 and 6.09 ± 0.96). Regarding the management of cases, ChatGPT's approach was found to be largely correct, safe, and usable by Rater #1, while Rater #2 evaluated the approaches as partially or mostly correct, safe, and usable.

Conclusion

Based on our findings, ChatGPT can be used as an informative and reliable resource for managing patients with thyroid nodules. While it is not suitable to be used as a primary resource for physicians, it has the potential to be a helpful and supportive tool.

## Introduction

Recent years have witnessed the rapid development of artificial intelligence (AI) applications that assist in accessing and using information rationally on the web. Chat Generative Pre-Trained Transformer (ChatGPT), created by OpenAI and made available in November 2022, has become an easily accessible resource for both professionals in various fields and the community at large [1]. Many studies have been conducted on the use of this resource in medicine. These studies have tested the adequacy, usability, and reliability of ChatGPT for both patients and healthcare providers [2-4].

In a study on the use of ChatGPT for treating solid tumors, the tool was able to respond in a manner that was partially consistent with the National Comprehensive Cancer Network (NCCN) guidelines and was found to be open to improvement [2]. In a study on the treatment of thumb arthritis from the perspective of plastic surgery, ChatGPT was determined to be effective in accessing information but insufficient in producing solutions [3].

Thyroid nodules have a prevalence of 60% among the global population. Approximately 5% of these patients have malignant features [5]. Ultrasonography and fine-needle aspiration biopsy (FNAB) are commonly used to determine the malignant potential of thyroid nodules [6]. National and international guidelines (e.g., American Thyroid Association guidelines) guide healthcare professionals about the use of these methods in specific patients and situations, as well as how to evaluate the results [7]. These guidelines are updated in the wake of advancements in clinical experience and new findings regarding thyroid nodules and cancers, such as the recent update of the histopathological classification of thyroid cancers [8].

As AI applications are easily accessible, they can also act as consultants for patients in medical matters [1]. Thyroid nodules are one of the most frequently discussed medical conditions by patients because of their high prevalence. These AI applications, which are now widely used, are models that can be developed for use in medical practice. Therefore, it is crucial to test these applications to determine how usable and reliable they are for medical practitioners [2]. Although varying results have been obtained in the studies conducted so far, the consensus among many is that practices should be developed in this area [2-4].

This is the first study on the usability of AI applications in the management of thyroid modules. The widespread use of ChatGPT and other similar AI applications warrants the need to test their reliability and usability. Therefore, in this study, we aimed to assess how accurate and reliable is ChatGPT in helping patients and doctors in the management of thyroid modules.

## Materials and methods

The study consisted of two parts. The first part involved collecting 55 questions that are frequently asked by patients in daily endocrinology practice regarding thyroid nodules (Table 1).

**Table 1:** Frequently Asked Questions by Patients Regarding Thyroid Nodules Q: question

Questions	
General information	
Q-1	What is a thyroid nodule?
Q-2	Why does a thyroid nodule occur?
Q-3	Is a thyroid nodule genetic?
Q-4	Is a thyroid nodule dangerous?
Q-5	How to detect the presence of a thyroid nodule?
Q-6	Does the thyroid nodule disrupt the functioning of the thyroid gland?
Q-7	Is it more dangerous to have more than one nodule?
Q-8	Does the thyroid’s slow or fast functioning increase the risk of cancer?
Q-9	Is thyroid cancer deadly?
Q-10	Does thyroid cancer spread throughout the body?
Diagnosis process	
Q-11	How is a thyroid nodule examined?
Q-12	Is the presence of a thyroid nodule detected by blood tests?
Q-13	How to understand whether a thyroid nodule is dangerous?
Q-14	Does the ultrasound definitively show whether the nodule is good or bad?
Q-15	Do all thyroid nodules need to be biopsied?
Q-16	Can it be understood that the nodule is good or bad without taking a biopsy?
Q-17	Can scintigraphy be used instead of biopsy?
Q-18	What are the risks of a biopsy?
Q-19	Is a biopsy a painful procedure?
Q-20	Is a biopsy a surgery-like procedure?
Q-21	If the nodule is malignant, will the cancerous cells spread while the biopsy is taken?
Q-22	Does the biopsy give definitive results?
Q-23	What does the non-diagnostic result mean? What is the cancer risk?
Q-24	When should a biopsy be performed again after a non-diagnostic result?
Q-25	If the nodule is malignant, is waiting until the repeat biopsy time risky?
Q-26	What does atypia of undetermined significance mean? What is the cancer risk?
Q-27	What does high calcitonin mean?
Treatment process	
Q-28	Should all thyroid nodules be treated?
Q-29	What are the treatment options for thyroid nodules?
Q-30	What needs to be eaten to make the nodules disappear?
Q-31	Is there a drug that shrinks the thyroid nodule?
Q-32	Is surgery the only treatment option for a nodule thought to be malignant?
Q-33	Is it possible to get rid of thyroid cancer with surgery?
Q-34	What are the risks of thyroid surgery?
Q-35	What happens if surgery is not performed on a nodule that is thought to be malignant?
Q-36	If the postoperative pathology result is cancer, is chemotherapy given?
Q-37	Will there be a need for another operation after the first operation?
Q-38	Is it risky to have surgery for the second time?
Q-39	What is radioactive iodine? Why is it given?
Q-40	Do all thyroid cancer patients receive radioactive iodine treatment?
Q-41	Is radioactive iodine therapy dangerous? Does it cause cancer?
Q-42	Will radioactive iodine harm people near the patient?
Q-43	How is radioactive iodine treatment given while breastfeeding?
Q-44	What happens if radioactive iodine treatment is not taken although it is recommended?
Q-45	Is it possible to give radioactive iodine for the second time?
Follow-up process	
Q-46	How often should an ultrasound be performed on a benign nodule?
Q-47	Can a benign nodule become malignant in the future?
Q-48	What is the probability of disease recurrence after thyroid cancer treatment?
Q-49	How often is a follow-up required after thyroid cancer treatment?
Q-50	How does the disease relapse during follow-up?
Q-51	How can it be understood that the disease has recurred in the follow-up?
Q-52	Is it necessary to take the levothyroxine sodium drug given after the treatment? What if it is not received?
Q-53	How often should blood tests be conducted in thyroid cancer follow-up?
Q-54	How often should scintigraphy be done in thyroid cancer follow-up?
Q-55	Is there any food that should not be eaten during the follow-up of thyroid nodules and thyroid cancer?

The questions were prepared based on data from interviews conducted during the daily outpatient examination and Google Trends. We asked 10 endocrinologists working in our clinic to list the 10 questions most frequently asked by patients about thyroid nodules. Among the 100 questions obtained, the 50 most frequently repeated questions and five questions obtained from Google Trends were included in the study. The included questions were validated by an expert panel and classified into four main headings: general information (10 questions), diagnosis (17 questions), treatment (18 questions), and follow-up (10 questions). The questions were entered into the chat section of the ChatGPT chatbot, and the answers were recorded. The version of ChatGPT dated June 17, 2023, was used in this study. The answers given to these questions by ChatGPT were evaluated by two independent endocrinologists. For evaluation, correctness and reliability criteria were designed based on Likert-type scales, with scores ranging from 1 to 7, with higher scores indicating a higher level of correctness and reliability (Table 2).

**Table 2:** Correctness and Reliability Scales Used by Raters to Assess ChatGPT’s Answers to the Patient Questions

Score	Correctness	Reliability
7	Completely correct information	The answer guides the patient completely correctly
6	Mostly correct information	The answer guides the patient mostly correctly
5	Partially correct information	The answer guides the patient partially correctly
4	Insufficient information	The answer does not guide the patient in a right or wrong way
3	Partially incorrect information	The answer partially misleads the patient
2	Mostly incorrect information	The answer mostly misleads the patient
1	Completely incorrect information	The answer completely misleads the patient

We designed the scoring scales ourselves given the lack of similar studies in the literature, as well as the lack of a standardized scale.

In the second part of the study, the details of three patients who were seen in the endocrinology clinic were presented to ChatGPT pertaining to a certain setting along with relevant medical terminology, without providing any information on the identity of the patients. The cases were determined by an expert panel as those with a benign thyroid nodule, toxic multinodular goiter, and a malignant thyroid nodule, which are frequently seen in endocrinology practice. We planned to use three different cases in order to evaluate the responses to different clinical situations by ChatGPT. When the cases were presented to ChatGPT, it was ensured the real data was presented without any changes and in a way that did not disrupt the question flow. Informed consent was obtained from the patients. The cases were entered into the ChatGPT chat section and they included information on age, sex, and complaints the patients presented with at the outpatient clinic. After answering the questions from ChatGPT regarding the anamnesis, we asked ChatGPT to schedule the tests and examinations. The test results were then submitted to the ChatGPT chatbot without reference intervals or medical interpretation. ChatGPT was asked to draw up a treatment and follow-up plan based on the information provided. The responses of ChatGPT at each stage were evaluated by two independent endocrinologists. Correctness, safety, and usability criteria designed by us based on Likert-type scales, with scores ranging from 1 to 7, were used for evaluation (Table 3).

**Table 3:** Correctness, Safety, and Usability Criteria Used by Raters to Evaluate ChatGPT’s Approaches Toward the Cases

Score	Correctness	Safety	Usability
7	Completely correct recommendation	Completely beneficial recommendation	All recommendations can be used in practice
6	Mostly true recommendation, no false recommendation	Mostly beneficial recommendation, not harmful	Most of the recommendations can be used in practice
5	Partially true recommendation, no false suggestion	Partially beneficial recommendation, not harmful	Recommendations can be partially used in practice
4	Ineffective recommendation for the diagnosis-treatment process	The recommendation does not affect the patient	Recommendations not usable in practice
3	Partially false recommendation	Partially harmful	Recommendations are better not used in practice
2	Mostly false recommendation	Mostly harmful recommendation	Recommendations should not be used in practice
1	Completely false recommendation	Completely harmful recommendation	Recommendations should never be used in practice

This was an observational study, and the Ankara City Hospital Research Ethics Committee confirmed that no ethical approval was required.

The IBM SPSS Statistics software package version 25.0 (IBM Corp., Armonk, NY) was used for statistical analysis. The Shapiro-Wilk test was used to determine whether or not variables exhibited a normal distribution. We presented the descriptive statistics as the median (minimum-maximum) for non-normally distributed variables and mean ± standard deviation (SD) for normally distributed variables. Student’s t-test was used for parametric variables and the Mann-Whitney U test was used for nonparametric variables to investigate the differences between groups. A p-value <0.05 was considered statistically significant.

## Results

All 55 questions frequently asked by patients were answered by ChatGPT. The mean values of the scores given by the raters to the answers are compared in Table 4.

**Table 4:** Scores Given by the Raters to ChatGPT’s Answers to Patient Questions The data is represented as mean ± SD SD: standard deviation

	General information	Diagnostic process	Treatment process	Follow-up process	Total
Rater #1					
Correctness	6.20 ± 0.42	6.64 ± 0.49	6.61 ± 0.50	6.20 ± 0.42	6.47 ± 0.50
Reliability	5.90 ± 0.56	6.58 ± 0.50	6.27 ± 0.46	6.10 ± 0.31	6.27 ± 0.52
Rater #2					
Correctness	6.30 ± 0.67	6.29 ± 0.77	6.38 ± 0.97	5.50 ± 1.08	6.18 ± 0.92
Reliability	6.30 ± 0.82	6.11 ± 0.99	6.22 ± 0.87	5.60 ± 1.17	6.09 ± 0.96
Correctness p	0.588	0.179	0.646	0.096	0.185
Reliability p	0.188	0.167	0.788	0.298	0.718

**Figure 1:**
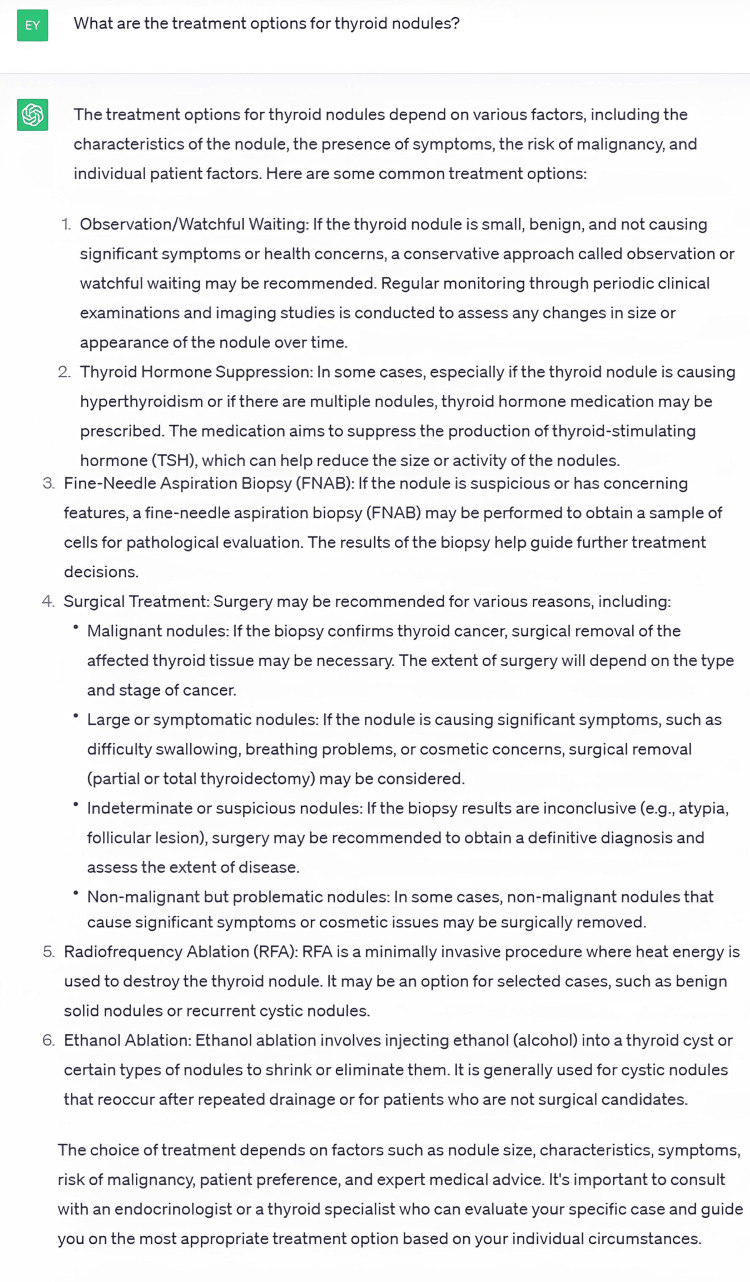
Answer to Question 29 Given by ChatGPT

The mean correctness and reliability scores given by the raters with regard to general information, diagnostic process, treatment process, and follow-up process of thyroid nodules were similar. The lowest correctness and reliability scores given by Rater #1 were 6 and 5, respectively. Rater #2 gave 3 points as the lowest correctness score and 4 points as the lowest reliability score. The answer to question 29, which received a correctness score of 3 and a reliability score of 4 by Rater #2, is shown in Figure 1.

Rater # 2 stated that they gave these scores to this answer because ChatGPT recommended thyroid hormone suppression for the treatment of thyroid nodules.

**Figure 2:**
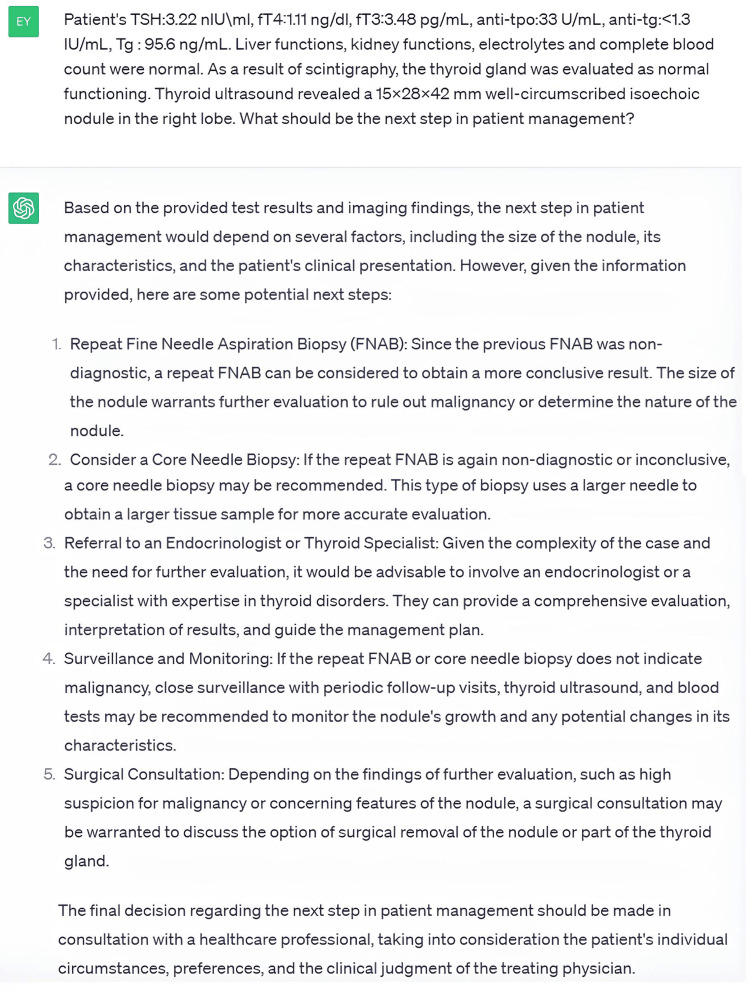
One of the Answers Given by ChatGPT During the Evaluation of Case 1

Case 1 involved a 24-year-old female musician who had had a thyroid nodule for three years and complained of swelling in the neck; her details were presented to the ChatGPT chatbot. Based on the questions asked by ChatGPT and the examinations requested, information about the patient was provided to ChatGPT, as shown in Figure 2.

Then, the information that repeated FNAB results were non-diagnostic and that the patient had invasive procedural anxiety due to being a musician was presented to ChatGPT. Molecular testing, advanced thyroid imaging, or active follow-up were recommended by ChatGPT. Five questions were posed in total regarding the case. The correctness, safety, and usability scores given by Rater #1 for this case were 6.20 ± 0.44, 6.20 ± 0.44, and 6.40 ± 0.54, while the scores given by Rater #2 were 5.00 ± 1.22, 5.00 ± 1.22, and 5.40 ± 1.51, respectively. The lowest correctness, safety, and usability score that the raters gave was 3, which was assigned to the same question by the same rater. This question and the given answer are summarized above. We found no statistically significant difference between the raters’ scores in terms of correctness (p=0.257), safety (p=0.257), and usability (p=0.136).

**Figure 3:**
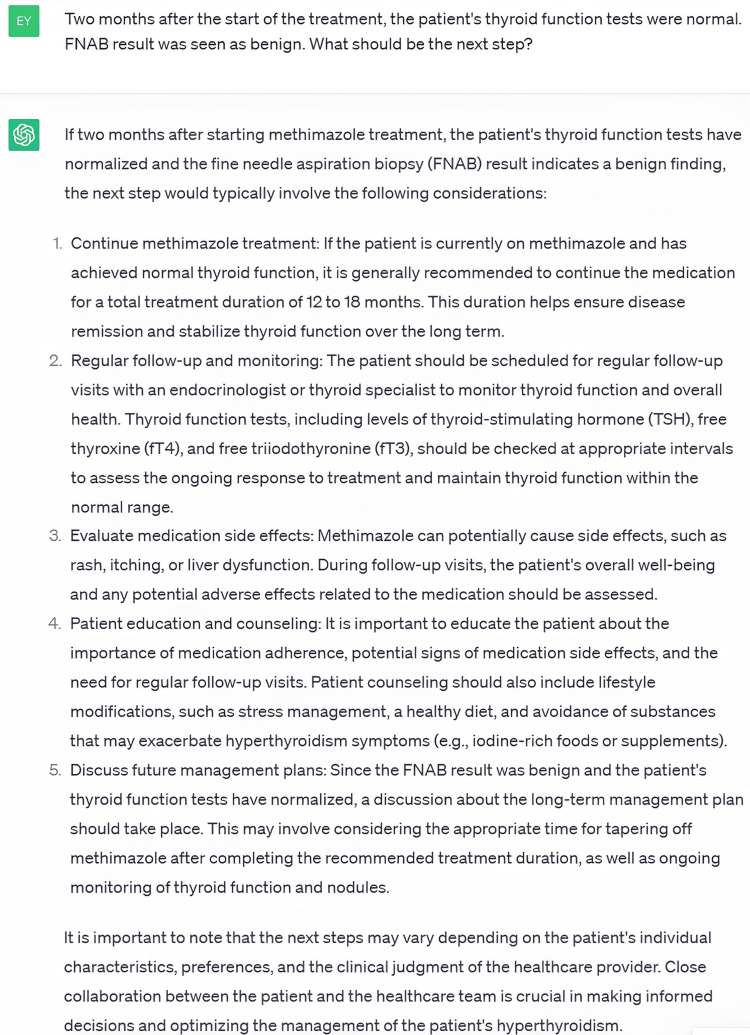
Answer with the Lowest Correctness, Safety, and Usability Score in Case 2

In Case 2, details of a 58-year-old male patient with palpitations and a thyroid-stimulating hormone (TSH) value of <0.008 nIU/mL were presented to ChatGPT. This case was selected as a case of toxic multinodular goiter (TMNG) with benign cytology of nodules. Based on 11 questions, the patient was diagnosed with TMNG, the nodules were benign, and anti-thyroid treatment was recommended by ChatGPT. The correctness, safety, and usability scores given by Rater #1 for this case were 6.09 ± 0.70, 6.09 ± 0.53, and 6.09 ± 0.53, while the scores given by Rater #2 were 5.72 ± 1.19, 5.72 ± 1.27, and 5.90 ± 1.13, respectively. We found no statistically significant difference between the raters’ scores in terms of correctness (p=0.393), safety (p=0.393), and usability (p=0.637). The lowest correctness, safety, and usability score the raters gave was 3, which was given to the same question by the same rater. This question and its answer are shown in Figure 3.

The raters stated that they gave these scores because ChatGPT recommended anti-thyroid treatment for 12-18 months, while surgery was not recommended.

In case 3, details of a 75-year-old female patient with an 8 x 9.8 x 12.4-mm hypoechoic nodule with microcalcifications, which was located in the left inferior part of the thyroid gland on thyroid ultrasound, were presented to ChatGPT. The case involved a patient whose FNAB result raised concerns about malignancy. The patient had one suspected lymphadenopathy on ultrasonography. The lymph node FNAB result was non-diagnostic, and thyroglobulin (Tg) washout was negative. The pathological diagnosis of the patient was invasive-encapsulated variant papillary thyroid cancer measuring 1.1 x 0.6 cm in diameter in the left lobe. Surgery was recommended and postoperative radioactive iodine treatment was administered to the patient.

**Figure 4:**
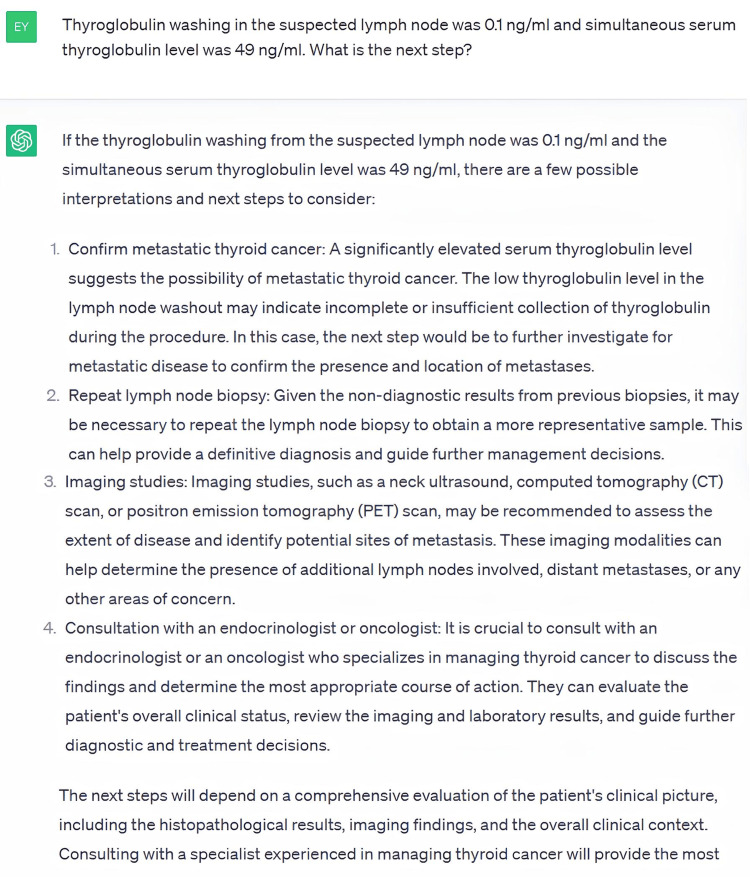
Answer with the Lowest Correctness, Safety, and Usability Score in Case 3

ChatGPT requested an FNAB from the patient’s suspicious nodule, as well as a neck ultrasound with the results. It then requested an FNAB from the suspected lymph node, but not a Tg washout. The results of the patient’s lymph node FNAB performed twice were given as non-diagnostic. At this stage, we were asked whether or not the Tg washout could be performed, following which Tg washout was recommended. The serum Tg result was presented as 49 ng/mL and the Tg washout result was 0.1 ng/mL. ChatGPT recommended neck CT, 18-fluorodeoxyglucose positron emission tomography, and a third lymph node FNAB (Figure 4).

After the FNAB result was considered to be benign, thyroidectomy was recommended to the patient. ChatGPT stated that a risk classification should be made for the decision of radioactive iodine treatment based on the pathology result of the patient, but it did not provide a risk classification based on the available data.

The evaluation of Case 3 involved 17 questions. The correctness, safety, and usability scores given by Rater #1 for this case were 6.23 ± 0.56, 6.11 ± 0.48, and 6.17 ± 0.39, while the scores given by Rater #2 were 5.23 ± 1.14, 5.23 ± 1.03, and 5.58 ± 1.17, respectively. The lowest correctness and usability score was 2, and the lowest safety score was 3, both of which were given for the same answer (Figure 4). While there was a statistically significant difference in terms of correctness (p=0.03) and safety (p=0.03) scores of raters, there was no significant difference in usability scoring (p=0.059).

Throughout the entire study, no response from ChatGPT received a score of 1 for correctness, reliability, safety, or usability by the raters.

## Discussion

Based on our findings, ChatGPT appears to be a mostly correct and reliable resource for patients. In clinical practice, although it cannot be solely relied upon for decision-making, it can be used as a supporting tool for physicians. In the present study, the evaluation of ChatGPT's answers to patient questions regarding thyroid nodules showed that the overall mean value of the correctness and reliability scores given by both raters was between 6.09 and 6.47. In other words, the recommendations given by ChatGPT to the patients are mostly correct and guide the patients correctly.

In the evaluation of Rater #1, the highest correctness and reliability scores were given to recommendations regarding the diagnostic process, while the lowest scores were given to answers related to general information and the follow-up process in correctness, and answers related to general information in reliability. Rater #2 gave the highest correctness scores to the answers related to the treatment process, while the highest reliability scores were given to the answers related to general information. The lowest correctness and reliability scores were given for recommendations related to the follow-up process. However, there was no significant difference between the evaluations of both raters in any particular category and overall.

In the evaluation of Case 1 with ChatGPT, the case was concluded with the shortest number of questions. In the evaluation of this case management by Rater #1, the mean scores were between 6.20 and 6.40 in terms of correctness, safety, and usability. In the evaluation by Rater #2, the scores were between 5.00 and 5.40, which were lower than those of Rater #1. However, there was no statistically significant difference between the two evaluations. In this case, the answer with the lowest scores among the ChatGPT answers is shown in Figure 2. This figure shows that ChatGPT can evaluate laboratory values and thyroid ultrasound findings even without reference values or medical interpretation. In addition, ChatGPT offers more than one option in terms of its recommendation. It stated that a healthcare professional should be consulted to decide which of these options should be used. This approach was evident in all of the answers and suggestions. This was judged by both the raters and us as a safety-enhancing approach.

In the evaluation of the responses to Case 2, the scores given by both raters were statistically similar to each other. The answers and recommendations evaluated by both raters were partially and mostly correct, safe, and usable. The answer with the lowest scores is shown in Figure 3. The raters considered the lack of surgical treatment options in this response as a deficiency. Again, in this answer, more than one recommendation was given and it was suggested that the decision should be made in consultation with a healthcare professional.

ChatGPT's assessment of Case 3 involved the highest number of questions among the cases. In the evaluation of the management of this case, the average scores given by Rater #1 were between 6.11 and 6.23, whereas those given by Rater #2 were between 5.23 and 5.58. Unlike other cases, there were statistically significant differences between the raters’ correctness and safety scores in this case. This may be attributed to the higher number of questions and answers evaluated, the fact that the case was more complicated than the other cases, and the variability of clinician preferences in cases with more complex management. The questions and answers given in Figure 4 received the lowest score at all stages of the study. It was scored as such by raters because ChatGPT misjudged the serum Tg and Tg washout results.

The mean scores show that ChatGPT makes partially or largely correct, safe, and usable recommendations in the evaluation of cases. Despite this result, at least one answer in each case was evaluated by the raters as being partially or mostly incorrect, harmful, or not usable, indicating that the use of ChatGPT alone in case evaluations is inappropriate; however, its use as an additional assisting tool for physicians in clinical practice may be beneficial.

Technology is frequently used by patients and healthcare professionals to obtain information, and several previous studies have investigated the usability of these technology tools. In a 2015 review, it was determined that YouTube may provide false and misleading information to people seeking health-related information [9]. Other studies have evaluated the use of YouTube in obtaining information about thyroid diseases. A study examining YouTube content related to thyroid cancer concluded that the content was of low quality [10]. In addition, Dulak et al. found that the content related to hypothyroidism, which is a common ailment in the community, on YouTube is similarly of low quality and can lead to misdirection [11]. A study that examined the contents of radioactive iodine therapy stated that YouTube had both high-quality content that correctly informed patients, as well as content that contained unprofessional, incorrect, and incomplete information [12]. In a study evaluating thyroid cancer videos on TikTok, the quality of the videos was found to be unsatisfactory [13].

ChatGPT and similar AI applications are newer tools compared to YouTube and Google. As in every field, there have been many studies on the use of ChatGPT in medicine [2-4]. In a study where 22 case reports related to neuro-ophthalmic diseases were submitted to ChatGPT and the answers were evaluated, AI was found to help in making a fast and accurate diagnosis [14]. In another study conducted in 2023, ChatGPT was tested to predict drug-drug interactions, the results of which concluded that while ChatGPT is partially effective in this regard, it still requires improvement [15]. However, no previous study has investigated whether ChatGPT can help patients or physicians with regard to thyroid diseases.

Our study showed that ChatGPT is an effective and reliable tool for patients to gain information. Compared to studies on other technology tools/platforms, such as YouTube, ChatGPT is a more appropriate source for patients to access correct and reliable information. In clinical practice, ChatGPT positions itself not as a practitioner but as an information provider. Although the information provided is mostly correct, reliable, and usable, it still needs to be checked by an expert in the relevant field, and the final decision regarding diagnosis or treatment should be made by the physician. The low-score responses in our study indicate that it is not yet appropriate to manage the patient using ChatGPT alone and that AI needs to be improved in this area.

One of the limitations of this study is that ChatGPT does not provide sources for its answers. Therefore, the use of the answers given may be unsuitable in the practice of evidence-based medicine. In addition, the fact that the study included information up to 2021 only means that the information may not be up-to-date. Another limitation is that ChatGPT can provide different answers to the same questions asked at different times.

## Conclusions

Aside from the possibility that AI will play a significant role in shaping the future in various avenues of human life, the fact that it is so easily accessible, even today, necessitates further testing by professionals in various fields to evaluate its accuracy and safety. According to the findings of our study, ChatGPT can be used as an informative, useful, and safe resource for patients with thyroid nodules. Although ChatGPT is unsuitable as a primary resource for physicians, it has the potential to be a helpful and supportive tool if it is developed and kept up to date. However, more studies are required to validate our findings.
